# Long-term effect of chemotherapy after ovarian decortication on the ovarian function in women surviving cancer

**DOI:** 10.1007/s10815-023-02949-y

**Published:** 2023-09-27

**Authors:** Ester Ortiz, Carlos J. Peña, Juan-José Hidalgo, Aitana Monllor-Tormos, Irene Zolfaroli, María-José Vila, María Sánchez-Serrano, Antonio Pellicer, Antonio Cano

**Affiliations:** 1 Service of Obstetrics and Gynecology, University Hospital Dr Peset, Av Gaspar Aguilar 90, 46017 Valencia, Spain; 2 Bioinformatics and Biostatistics Unit, INCLIVA, Calle Menéndez Pelayo 4, 46010 Valencia, Spain; 3 Department of Gynecologic Oncology, La Fe University and Polytechnic Hospital, Av Fernando Abril Martorell 106, 46026 Valencia, Spain; 4 Service of Obstetrics and Gynecology, INCLIVA, Av Blasco Ibáñez 19, 46010 Valencia, Spain; 5 IVI RMA Rome Rome Italy; IVI Foundation, Instituto de Investigación Sanitaria La Fe (IIS La Fe), Valencia, Spain; 6 Department of Pediatrics, Obstetrics and Gynecology, University of Valencia, INCLIVA, Av Blasco Ibáñez 15, 46010 Valencia, Spain

**Keywords:** Ovarian decortication, Fertility preservation, Recovery, Anti-Müllerian hormone, Menstrual pattern

## Abstract

**Purpose:**

Ovarian decortication may affect ovarian function. We investigated the status of ovarian reserve after ovarian decortication plus chemotherapy at a stage of presumed stabilized recovery in women surviving cancer.

**Methods:**

We searched our database for cancer survivors subjected to ovarian decortication and chemotherapy at least 3 years previously. Ovarian function was explored for levels of anti-Müllerian hormone (AMH), follicle-stimulating hormone (FSH), and estradiol (E2), and menstrual pattern.

**Results:**

Forty women (mean age 29.6 (SD, 6.1) years) were assessed at a mean of 4.7 (1.5) years after surgery. The predecortication levels of AMH and FSH changed at post-treatment from 2.2 (1.4) to 0.5 (1.3) ng/mL for AMH (*p* < 0.001) and from 4.7 (2.1) to 16.7 (21. 6) IU/L for FSH (*p* < 0.001). Amenorrhea consistent with primary ovarian insufficiency (POI) was diagnosed in 11 women, and normal ovarian reserve (AMH ≥ 1.0 ng/mL) was found in 4 of the 21 women who recovered regular cycles. Logistic regression confirmed AMH as an independent predictor of diminished ovarian reserve (OR = 0.24, 95% CI: 0.04-0.63, *p* = 0.025) and POI (OR = 0.11, 95% CI: 0.01–0.52, *p* = 0.027), and age was predictive of POI (OR = 1.36, 95% CI: 1.08–1.96, *p* = 0.035) and of irregular menstrual cycle (OR = 1.20, 95% CI: 1.03–1.46, *p* = 0.034).

**Conclusion:**

Ovarian decortication plus chemotherapy had a deleterious effect when assessed at a stage of stabilized ovarian recovery, but whether ovarian decortication had a specific impact cannot be revealed from our data.

## Introduction

Modern treatments have improved survival rates of young women with cancer. These therapies, however, may have a gonadotoxic effect, with a detrimental impact on ovarian function and fertility. The size of the effect depends not only on the type of the drug but also on individual patient variations. Evidence from previous studies has identified age, cancer type, and pretreatment anti-Müllerian hormone (AMH) as predictors of ovarian performance after use of gonadotoxic drugs in chemotherapy [1, 2].

Recent years have seen the development of different fertility preservation (FP) procedures, among which cryopreservation of embryos or oocytes, pharmacological protection with gonadotropin releasing hormone analogs (GnRHa), ovarian transposition, or ovarian tissue cryopreservation (OTC) have been used with variable success [3–7].

OTC has been selected as an alternative to mature oocyte cryopreservation when ovarian stimulation protocols are not an option [8], such as in urgent chemotherapy or in prepubertal patients. Implanting cryopreserved ovarian tissue is a reasonably consolidated practice worldwide. A recent data review from 5 renowned European centers found over 75 women with one or two live births after cryopreserved ovarian tissue implantation, this number rising to more than 200 cases on a global level [9].

The dynamics of ovarian function recovery after gonadotoxic treatment completion seems to affect biomarkers of ovarian reserve like follicle-stimulating hormone (FSH) and AMH differently. Moreover, time to stabilization during the recovery process is unclear, and according to recent data might take up to 3–4 years for AMH [10].

Ovarian decortication, the surgical process consisting of extracting ovarian cortex and keeping it frozen until re-implantation if needed, may have a different impact on post-chemotherapy ovarian function because the excision process already imposes a reduction in ovarian reserve prior to gonadotoxic treatment. How prognosis in terms of ovarian function recovery and fertility may change is an important yet understudied issue [11]. The aim of this study was to evaluate the impact of chemotherapy on ovarian reserve as measured by AMH, FSH, and estradiol (E2) values in a cohort of cancer patients previously undergoing fertility-preserving ovarian decortication. In addition, we selected a time interval (average 4.7 years postoperatively) in which ovarian recovery was likely to be stabilized.

## Materials and methods

Our OTC program was open to women from the Oncology Department of our hospital and from other referral centers nationwide. On arrival, women completed a detailed anamnesis comprising menstrual data and pregnancy history, medical conditions, lifestyle factors, and potential infertility. A clinical assessment was performed, including anthropometric parameters (height and weight), pelvic examination, and endo-vaginal ultrasound to explore the uterus and ovaries. Blood samples were taken between 08.00 and 10.0 h after 12-h fasting. The analytical study included parameters as per protocol for surgical intervention under general anesthesia, together with hormonal measurements. Premenopausal status was confirmed by a regular menstrual cycle together with premenopausal hormonal levels of FSH (range 2.4–6.6 IU/L) and E2 (range 21–251 pg/mL), both in the follicular phase.

Ovarian decortication was performed by laparoscopy using a previously described technical procedure [12]. Briefly, the cortex from one of the ovaries was removed with scissors, and the hemostasis of exposed medullary tissue was ensured with electrocautery. The extracted ovarian cortex was placed in Ham F10 medium and cut into strips whose thickness did not exceed 2 mm, to allow the action of the cryoprotectants.

Our database was searched for women who were premenopausal at the time of surgery (≤ 38 years) living in the metropolitan area of Valencia. The two inclusion criteria, obtained from their corresponding electronic medical record (EMR), were an interval of at least three years since surgery and completed chemotherapy schedule. Exclusion criteria were recent (≤ 4 weeks) use of any hormonal drugs, pregnancy or lactation, menstrual irregularities prior to decortication or a history of any pathology other than cancer linked with ovarian dysfunction, such as polycystic ovarian syndrome, hyperprolactinemia, thyroid disease, Cushing disease, infertility for at least 6 months, a previous diagnosis of a disease linked with premature ovarian insufficiency (POI), or another previous cancer diagnosis, with or without chemotherapy or radiotherapy.

Patients meeting the conditions were sent a letter of invitation to participate. Respondents were scheduled an appointment at our service, in which the objective of the study was explained and additional clinical information was retrieved, including the status of the malignant disease from EMR, accessible through the platform that interconnects all public hospitals in our autonomous region.

The clinical and analytical assessments prior to decortication were repeated in this post-treatment visit, rescheduling patients with regular menstruation who were not at the follicular phase (days 2–5) of the cycle. Menstrual data were categorized into regular (all cycles between 23 and 35 days), irregular if outside this range, and amenorrhea if no bleeding during the last 6 months. Diminished ovarian reserve (DOR) was defined when AMH values were < 1.0 ng/mL [2], and POI in the presence of amenorrhea, elevated serum FSH levels of > 35 IU/L, and age < 40 years. Occurrence of any previous pregnancies and presence of menopausal symptoms were also recorded.

**Fig. 1:**
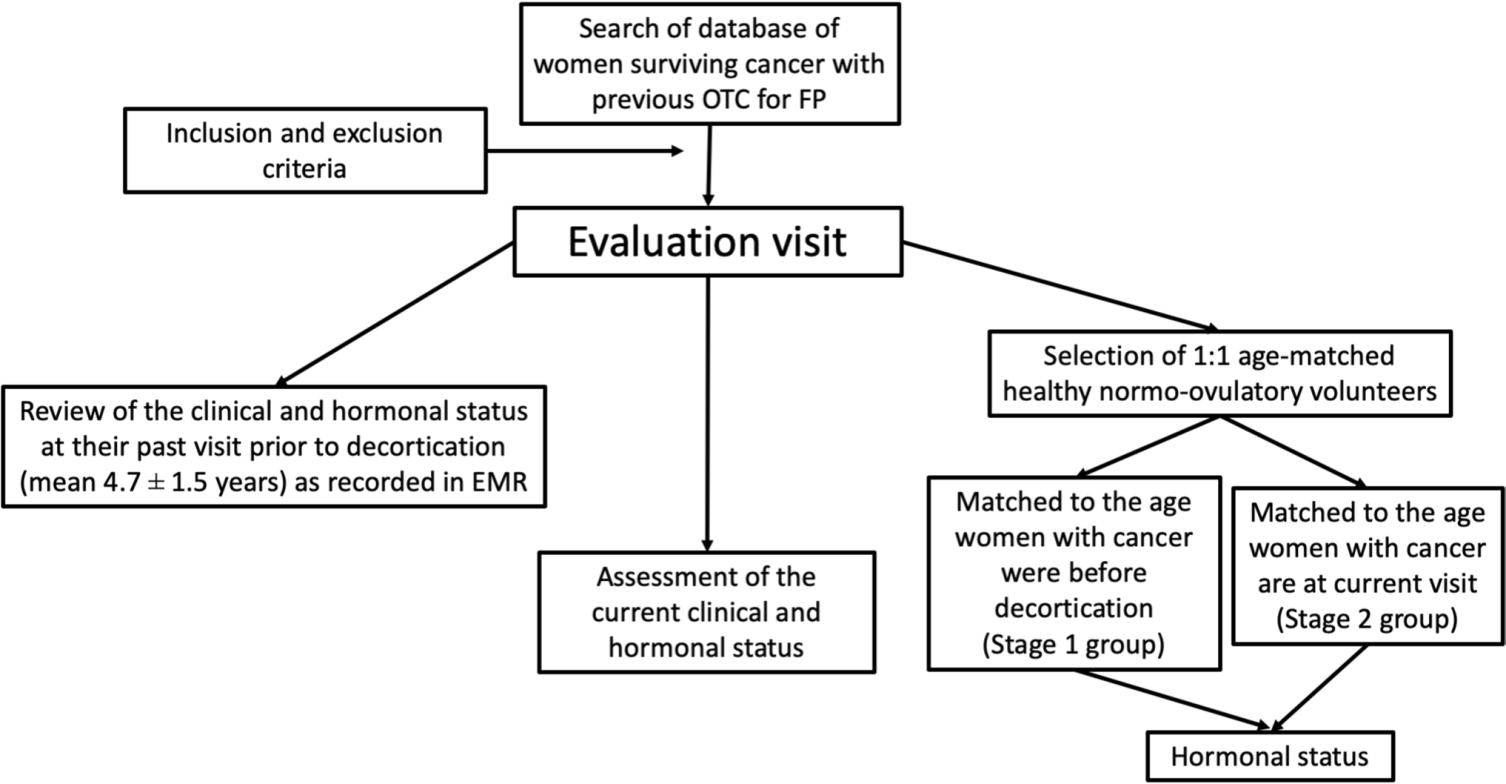
Women with previous cancer, who had undergone ovarian decortication for fertility preservation, were invited to participate in the study and evaluated at our center (evaluation visit). Current clinical and hormonal parameters were measured. In this step, predecortication clinical and hormonal parameters were retrieved from the EMR. In addition, two groups of volunteers with regular menstruation were recruited. These women were age-matched 1 : 1 to the cancer group at each stage, prior to decortication (stage 1, *n* = 40) and at the assessment visit (stage 2, *n* = 39, as one woman withdrew consent after enrollment). EMR: electronic medical record; FP: fertility preservation; OTC: ovarian tissue cryopreservation

Two cohorts of normal-ovulatory healthy women were matched 1 : 1 to the age the women with cancer were at the predecortication stage (stage 1 group, *n* = 40) and at the post-treatment visit (stage 2 group, *n* = 40). The purpose was to verify that women had normal values of AMH before starting treatment (predecortication, stage 1) and what was the impact of cancer treatment on ovarian reserve (stage 2), since the comparison with healthy normo-ovulatory coetaneous females in the post-treatment stage would reveal the confounding effect of age. Figure 1 shows the design of the study, which was approved by the Research Ethics Committee of our center (approval code 44.12). Informed written consent was obtained from all participants.

### Analytical assessment

Time-resolved chemiluminescent microparticle immunoassay (ARCHITECT, Abbott Laboratories) was used for assessment of FSH (analytical sensitivity 0.05 IU/L) and E2 (analytical sensitivity ≤ 10 pg/mL) in serum. Serum levels of AMH were analyzed with specific ELISA kits according to manufacturer’s instructions (AMH Gen II ELISA, Beckman Coulter, Inc., analytical sensitivity: 0.05 ng/mL).

### Statistical analysis

The Chi-square test of independence was performed to assess the relationship between categorical clinical variables (alcohol intake, smoking, obstetric history, previous use of hormonal contraceptives, post-cancer gestation, POI, and menstrual cycle pattern) and cancer type (breast cancer vs. Hodgkin’s lymphoma). For continuous clinical variables (age, body mass index, and menarche), Student’s *t*-test was carried out to study mean differences in both cancer groups. In case of deviations from the assumption of normal distribution according to Shapiro’s test, the non-parametric Wilcoxon rank-sum test was used.

To study the change in the mean hormone levels (AMH, FSH, and E2) before and after chemotherapy treatment in the cancer cohort, we used paired samples *t*-test or Wilcoxon signed-rank test. For healthy normo-ovulatory women, an independent *t*-test (or Wilcoxon rank-sum test) was applied as neither prechemotherapy nor post-chemotherapy groups were dependent. Finally, logistic regression was used to identify the independent predictors related to binary outcomes, DOR, POI, or menstrual cycle.

Results were considered statistically significant when *p* values fell below the 5% significance level. All statistical analyses were performed using the R software (version 4.2.2).

## Results

### Clinical and hormonal data

**Fig. 2:**
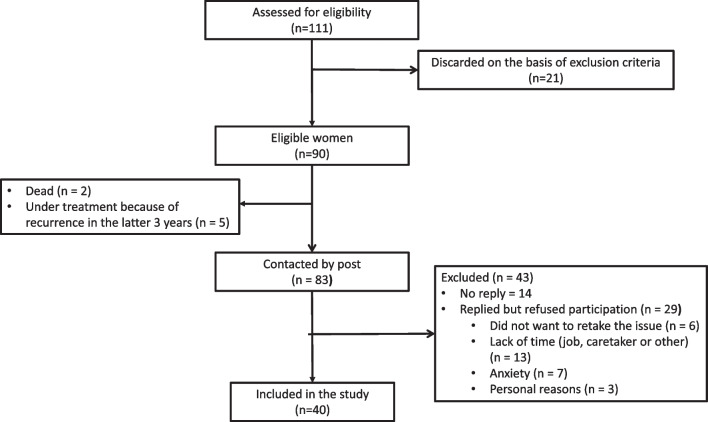
Flowchart diagram showing the number of women exposed to cancer who were assessed for eligibility, those remaining at each subsequent step of the study, and the reasons for withdrawal

Figure 2 shows the study flowchart. Our database search identified 111 women, of whom 90 met the eligibility criteria. A review of their EMR confirmed that two subjects had died and that five had suffered a recurrence during the last 3 years. After further exclusions, 40 women accepted to participate and were included in the study.

One of the 40 women in stage 2 post-treatment normo-ovulatory group withdrew consent for blood sampling, reducing the group to 39 participants.

The cancer-exposed cohort comprised 27 breast cancer patients, 12 with Hodgkin’s lymphoma, and 1 with adrenal carcinoma. All women had received chemotherapy, including alkylating drugs in all except 8 of those with Hodgkin’s lymphoma. Total body irradiation was applied to 3 Hodgkin’s lymphoma patients.

Clinical and hormonal characteristics at predecortication are presented in Table 1. Eight women were smokers, and 15 women were alcohol drinkers, but only at social gatherings. Nine women were past users of hormonal contraceptives. The mean (SD) age of women differed clearly by cancer type, at 32.2 (4.2) years in women with breast cancer and 24.5 (5.6) years in women with Hodgkin’s lymphoma (*p* < 0.001). The post-chemotherapy control was performed at a mean of 4.7 years (range 3–9 years) after surgery. Thirteen women were taking tamoxifen as adjuvant therapy for breast cancer, and 4 became pregnant spontaneously after completing chemotherapy, 2 miscarried and 2 delivered healthy babies.

**Table 1:** Clinical and hormonal characteristics at predecortication in cancer women (*n* = 40)

	Mean	Standard deviation
Age (years)	29.6	6.1
BMI (kg/m^2^)	22.8	3.3
Menarche (years)	12.1	1.3
AMH (ng/mL)	2.2	1.4
FSH (IU/L)	4.7	2.1
E2 (pg/mL)	50.1	24.3

*AMH* anti-Müllerian hormone, *BMI* body mass index, *E2* estradiol, *FSH* follicle-stimulating hormone

Prechemotherapy levels of ovarian markers were similar in women exposed to cancer and matched normo-ovulatory women (stage 1 group) for AMH (2.2 (1.4) ng/mL in cancer vs. 2.3 (1.5) ng/mL in healthy women) and E2 (50.1 (24.3) pg/mL in cancer vs. 46.3 (22.4) pg/mL in healthy women), but FSH values were slightly lower in the cancer group (4.7 (2.1) IU/L in cancer vs. 5.6 (1.5) IU/L in the healthy women (stage 1 group), *p* = 0.007). No hormonal differences were found between the breast cancer and Hodgkin’s lymphoma sub-cohorts.

### Effect of chemotherapy

**Fig. 3:**
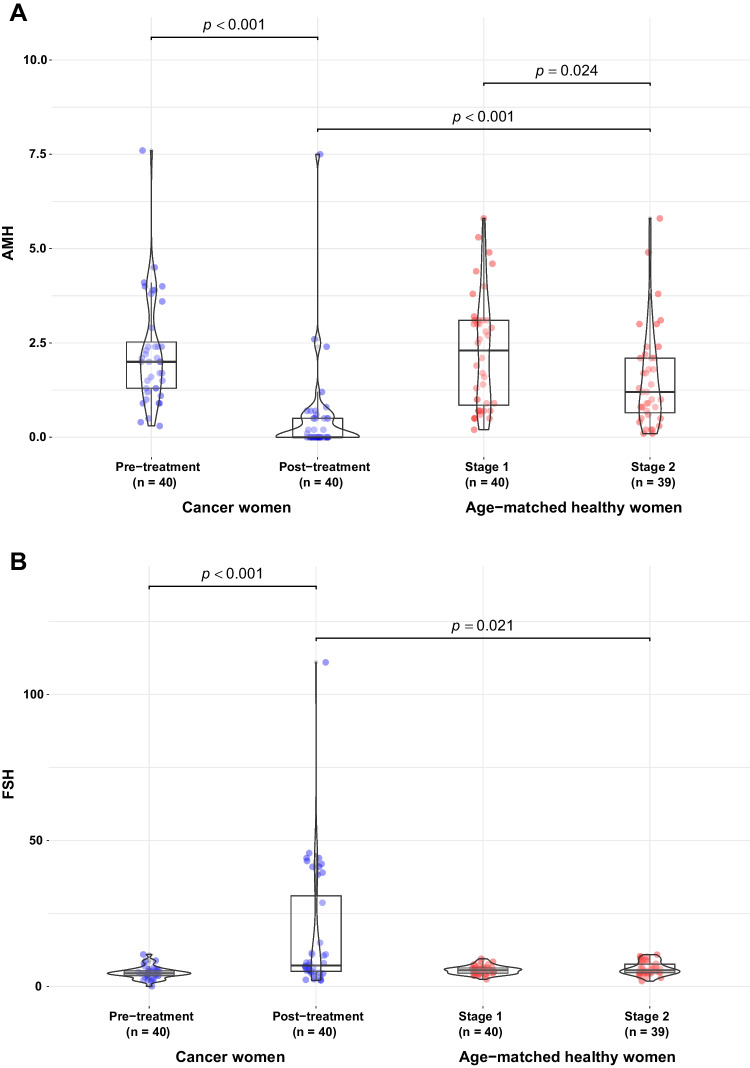
Box plots and violin plots for the visualization of the distribution of AMH (**A**) and FSH (**B**) levels across cohorts and treatment stage. The age-matched normo-ovulatory women includes stage 1, in which women were age-matched with cancer women at predecortication, and stage 2, in which women were age-matched with the cancer group at the post-treatment evaluation visit. AMH: anti-Müllerian hormone; FSH: follicle-stimulating hormone

Figure 3 shows the degree of change in AMH (A) and FSH (B) hormone levels induced by chemotherapy compared to the effects of age, as reflected by the corresponding levels in the healthy untreated (stage 2) women. Chemotherapy affected levels of both AMH, which decreased by 81.4% (*p* < 0.001) to 0.5 (1.3) ng/mL, and FSH, which increased by 318.2% (*p* < 0.001) to 16.7 (21.6) IU/L in the cancer exposed group. No significant change was found for E2.

There were significant differences between the post-chemotherapy cancer subjects and the age-matched healthy women (stage 2 group) for AMH, the 1.5 (1.3) ng/mL being significantly higher (*p* < 0.001), and for FSH, the 6.2 (2.3) IU/L being significantly lower (*p* = 0.021) in the stage 2 healthy women, while no difference was found for E2.

AMH levels were higher in the predecortication, stage 1 women than in the post-treatment heathy stage 2 group (2.3 (1.5) ng/mL vs. 1.5 (1.3) ng/mL, *p* = 0.024), probably reflecting the effect of age. No difference was found for FSH or E2.

Menstrual cycle regularity recovered in 54% (*n* = 21) of treated women: 13 women with breast cancer (48.1% of this sub-group) and eight (66.7%) women with Hodgkin’s lymphoma. Nonetheless, normal ovarian reserve (AMH ≥ 1.0 ng/mL) was preserved in only four women (10.0%). Recovery of regular menstrual cycles therefore co-occurred with normal ovarian reserve in only 19.0% of treated women. This was in clear contrast with the 22 women (56.4%) in the stage 2 healthy group (*p* < 0.001).

Amenorrhea consistent with POI was detected in 11 women (28.2 %), again with differences between tumor types, with nine women (33.3%) in the breast cancer group and two women (16.7%) with Hodgkin’s lymphoma. AMH levels were undetectable in all cases.

### Predictive variables

The predecortication variables affecting the probability of suffering DOR or POI were investigated with logistic regression (Table 2). AMH level was the only independent predictor of DOR (OR = 0.24, 95% CI: 0.04–0.63, *p* = 0.025), while age (OR = 1.36, 95% CI: 1.08–1.96, *p* = 0.035) and AMH (OR = 0.11, 95% CI: 0.01–0.52, *p* = 0.027) were predictors of POI. Age was also an independent predictor of menstrual irregularity (OR = 1.20, 95% CI: 1.03–1.46, *p* = 0.034).

**Table 2:** Results of the multivariable logistic regressions

Covariates	DOR			POI			Irregular menstrual cycle		
	OR	95% CI	*p* value	OR	95% CI	*p* value	OR	95% CI	*p* value
AGE at pretreatment	1.02	0.79, 1.28	0.85	1.36	1.08, 1.96	**0.035**	1.20	1.03, 1.46	**0.034**
BMI	0.94	0.67, 1.38	0.71	0.76	0.47, 1.09	0.20	1.05	0.82, 1.39	0.678
AMH at pretreatment	0.24	0.04, 0.63	**0.025**	0.11	0.01, 0.52	**0.027**	0.56	0.22, 1.17	0.158
Alcohol—weekends				0.57	0.04, 6.56	0.65			
Previous hormonal contraceptives				0.33	0.02, 3.61	0.38	0.66	0.09, 4.37	0.66

*CI* confidence Interval, *DOR* diminished ovarian reserve, *OR* odds ratio, *POI* primary ovarian insufficiency

Significant *p* values are in bold

## Discussion

Our study investigated the effect of chemotherapy on ovarian reserve and function in a cohort of oncological premenopausal women who had been subjected to ovarian decortication prior to chemotherapy for FP. Most cases in the cohort comprised two types of malignant tumors, breast cancer and Hodgkin’s lymphoma. This tumoral pattern reproduced the most prevalent tumor types found in larger series, such as Cobo et al.’s study of oocyte vitrification including 1073 cancer patients [13].

Cryopreservation of the ovarian cortex, a technique endorsed by scientific societies [8, 14–16], reduces the oocyte population prior to chemotherapy, this factor therefore potentially further decreasing ovarian reserve. A previous study investigated both ovarian reserve markers and menstrual patterns in women subjected to this technique, but only post-chemotherapy data were provided [17]. A small prospective study from the same authors confirmed that the decline in the circulating levels of AMH during the first week of chemotherapy was more pronounced in eight women subjected to unilateral ovariectomy than in nine whose ovaries were preserved [11]. This difference, although small, was maintained after the fourth cycle of chemotherapy. Subjects were then followed for up to one year after treatment completion. This preliminary observation agrees with results of studies of cystectomy due to endometrioma, which found an effect on circulating AMH levels [18, 19]. This background, together with the fact that our study subjects underwent decortication with the rest of the ovary preserved and received longer follow-up, indicate the importance of our study data about this far less frequent FP technique than oocyte freezing.

We designed a descriptive study with the objective of revealing the state of the ovarian reserve after cancer treatment in the stage in which it is fully recovered. The choice of this stage is important because otherwise the ovarian reserve could be underestimated. We selected three hormones (AMH, FSH, and E2) together with the menstrual status, as indicators of ovarian function at a mean of 4.7 years after surgery. A significant fall in AMH was found compared with the predecortication stage and age-matched healthy, stage 2 women (*p* < 0.001) (Fig. 3A). The reverse trend found in FSH was also altered significantly using the same comparators (*p* < 0.001 vs. predecortication, and *p* = 0.021 vs. stage 2 normo-ovulatory women) (Fig. 3B).

The dynamics of these changes cannot be extracted from our data, but previous evidence shows that the impact of chemotherapy on FSH and AMH levels begins early in both, stabilizing at around the fourth cycle of therapy (≈ 2 months) for AMH and slightly later, around the seventh cycle (≈ 3–4 months) for FSH [11]. In terms of recovery after chemotherapy, this seems to stabilize at the fourth month in the case of FSH [11]; according to a cross-sectional study, it occurs more slowly for AMH, requiring 2–4 years to reach a plateau, which is then maintained for 1–2 additional years before starting to decrease [10]. Our assessment at 4.7 years post-decortication seems therefore appropriate to avoid the unstable hormone stage.

The apparent variation in the recovery of the ovarian markers along the post-chemotherapy phase [10] means that the question of whether ovarian decortication accentuates ovarian function damage in the long term may only be answered by a prospective, head-to-head study. This is reinforced by the fact that published studies differ in key variables such as patient age, chemotherapy type, and most often in the availability of prechemotherapy hormonal levels. Moreover, data showing minor differences between women with and without ovarian excision in circulating AMH at only 4 weeks since chemotherapy initiation [11], together with results published in studies of patients with intact ovaries, which fall within the same range as our findings, favor the conclusion that the potential effect of ovarian decortication is minor compared to that of chemotherapy. In this regard, a study on a group of women with cancer who were younger (median age 28.3 years) than our cohort concluded that 79.4% met criteria for DOR at 8–24 months after therapy completion [2]. The effect is consistent with the 90% with DOR in our study. The post-treatment mean value of 0.52 ng/mL for AMH in our study is also comparable to the 0.81 ng/mL in another study also comprising younger women (median age 25.7 years) [20].

In our study, the pretreatment AMH levels were predictive of ovarian function recovery, as assessed by the number of women free of DOR, whereas age and AMH were predictive of POI. This information is consistent with previous findings [1, 2, 21] and with the well-established value of AMH and age as predictors of reproductive potential [22]. Therefore, good ovarian reserve, as deduced from high AMH levels or younger age, was the key factor in clinically evident recovery of ovarian function. In this regard, several clear strengths of OTC should be highlighted when considering fertility potential. The performance of the most frequently used technique, ovo-vitrification, has been well defined in a retrospective study using cryopreserved oocytes from oncological women [6]. Cumulative live birth rates reached 61.9% in women of ≤ 35 years, but this decreased to 43.4% in women > 35 years. The more limited published findings concerning ovarian cortex transplantation confirm that about one in four ovarian transplanted women give birth to a healthy child, according to data from 285 women treated in five European centers [9] and a systematic review [4]. Of interest, natural conception is also an option with this approach, reaching 30% success rates, rising in women who were younger at the time of OTC [9, 23]. Another advantage of OTC is its feasibility even in patients who have already received one or several cycles of chemotherapy [9], which is of interest as it allows OTC to be used in women with leukemia provided they have achieved complete remission, thus avoiding the risk of malignant cell reimplantation.

To conclude, our study provides information on ovarian function after a mean of 4.7 years in cancer survivors subjected to ovarian decortication and subsequent chemotherapy. Ovarian markers were available at the prechemotherapy stage. Our data seem to roughly overlap with previously published results in women without previous ovarian surgery. Therefore, although the hypothesis of a long-term effect of ovarian decortication on ovarian reserve recovery cannot be ruled out based on this study, our data give support to speculation that the impact, if any, seems small and, probably, lacks clinical significance. Translation of these data to potential fertility seems reassuring in the context of the success rates associated with OTC.

## Data Availability

Anonymized data will be made available upon request.
